# RE-Place: A Unique Project Collecting Expertise on New Approach Methodologies

**DOI:** 10.3389/fphar.2022.930148

**Published:** 2022-06-22

**Authors:** Mieke Van Mulders, Nancy Liodo Missigba, Birgit Mertens, Vera Rogiers

**Affiliations:** ^1^ SD Chemical and Physical Health Risks, Sciensano, Brussels, Belgium; ^2^ Faculty of Medicine and Pharmacy, In Vitro Toxicology and Dermato-Cosmetology, Vrije Universiteit Brussel (VUB), Brussels, Belgium

**Keywords:** 3Rs, replacement, alternatives to animal testing, open access, knowledge sharing, non-animal testing methods, New Approach Methodologies (NAMs), database

## Abstract

By applying “New Approach Methodologies (NAMs)” based on innovative technologies such as computer modeling, high throughput testing, omics, and sophisticated cell cultures, the use of experimental animals in the life sciences can be reduced or sometimes even completely avoided. Stimulating NAMs may benefit from a bottom-up approach, i.e., local initiatives mapping the available NAMs and promoting their use. An example of such an initiative in Belgium is the RE-Place project, which collects the available NAMs in one central database, and links this knowledge with the names of experts and research centers. To this extent, a template was created to collect the information of interest in a fast and consistent manner. Based on this template, a web-based application was developed to facilitate the entry of information, which was evaluated in a pilot study by experts in the field of NAMs. After integration of their feedback, a revised version of the RE-Place online tool was launched to the public. Aspects such as user-friendliness, quality of submitted information, protection of personal data and Intellectual Property (IP) rights were all considered in the development process. Hurdles like incentives for collaboration were also taken into account. Information submitted with the online tool is directly integrated in the RE-Place open access database. By consulting the database, scientists from various disciplines can easily identify the different types of NAMs and the experts using them in Belgium. As such, the RE-Place database contributes to building trust in the use of NAMs and stimulating their use and regulatory uptake.

## 1 Introduction

In 2016, the term ‘New Approach Methodologies’ (NAMs) was introduced for the first time during an international workshop of the European (EU) Chemicals Agency (ECHA) as follows: *“in silico approaches, in chemico and in vitro assays including high-throughput and high-content techniques, omics with a focus on metabolomics, the use of exposure data in terms of volume and use etc”* (ECHA, 2016)*.* This definition was later broadened by the ‘Interagency Coordinating Committee on the Validation of Alternative Methods (ICCVAM)’ to refer to (newly developed) methodologies that contribute to the replacement of animal testing. The definition: “*a broadly descriptive reference to any technology, methodology, approach, or combination thereof that can be used to provide information on chemical hazard and risk assessment that avoids the use of intact animals”* (ICCVAM, 2018). As NAMs are often based on the use of human body material, they are very valuable for the extrapolation of collected experimental data to human health.

At present, a number of NAMs are already fully integrated in the field of (regulatory) toxicology to assess the safety of chemicals, vaccines, medicines, cosmetic ingredients and other consumer products. Especially in Europe, due to the testing and marketing bans on finished cosmetic products and their ingredients, the use of NAMs became a routine practice in certain cases (European Parliament, 2009). Some of these have been transposed into official “Organisation for Economic Co-operation and Development Test Guidelines” (OECD TG) (OECD, 2004), which greatly facilitates their implementation for regulatory applications. This is especially true for local toxicity endpoints such as skin irritation and corrosion, eye irritation, sensitization and phototoxicity. However, for more complex endpoints such as systemic toxicity, the use of NAMs is in a less advanced stage. Although the term NAMs is mostly used in the field of (regulatory) toxicology, these methodologies can also be applied in several disciplines of fundamental and applied research. Given that most animals are used in biomedical sciences (European Commission, 2020), it is especially important to obtain a better overview of existing NAMs in this area.

Several initiatives for establishing reliable resources have already been launched to stimulate the use of NAMs, to facilitate knowledge sharing on 3Rs (Replacement, Reduction, Refinement), and to bridge the gap between regulators and biomedical scientists. Some of these have been launched by the “European Commission” (EC), the responsible authority in this area in Europe (Worth and Balls, 2001), such as the “Inventory of the 3Rs knowledge sources”^1^ and the “DataBase service on ALternative Methods” (DB-ALM)^2^. Other information resources include, amongst others, 1) the “ALTBIB,” developed by the National Library of Medicine, as a tool for scientists who are looking for information on alternatives to animal testing^3^, 2) “AnimAlt-ZEBET,” a database compiling methods linked to the 3Rs, published by the German Centre for the Protection of Laboratory Animals^4^, and ‘Norecopa’ a Norwegian platform to stimulate the 3Rs^5^.

Despite these initiatives, a report of the “EU Reference Laboratory for alternatives to animal testing” (EURL ECVAM) highlighted that more efforts are needed to increase knowledge sharing on 3Rs and improve access thereof (Roi and Kolar, 2019) (Holley et al., 2017). More specifically, the report indicated that, in general, scientists do not have time to study and review all available knowledge resources on 3Rs, and if they do, they are reluctant to use them as it remains often unclear whether the provided information is reliable and up-to-date (Roi and Kolar, 2019) (Holley et al., 2017). Centralizing the available information, and keeping it updated, would thus be a step in the right direction. Within this context, research projects, at regional, national and international level, could play an important role as they are often coordinated by experts from different disciplines and allow collaboration between sectors such as industry and academics (Holley et al., 2017).

An example of such a recent and regional research initiative is the “RE-Place project”^6^. This project was launched by the Flemish government in 2017, and 1 year later, the Brussels Capital region joined this initiative. The scope of the RE-Place project is threefold: 1) to obtain a clear and up-to-date overview of the available expertise on NAMs at a national level, 2) to raise awareness about the use of NAMs, and 3) to stimulate the further use and development of NAMs by improving the dissemination of information between relevant stakeholders (researchers, regulators, government, scientific institutions, industry, ethical committees, animal welfare bodies, and the public). The unique character of this project is that the different types of NAMs are linked with the names of experts and/or (research-) institutes, so that scientists who want to learn more about a particular NAM have direct access to the contact details of a relevant expert in Belgium.

One of the first steps was to clearly define the type of methodologies targeted by the RE-Place project. Based on discussions with the governmental responsibles and other national experts in the field of 3Rs, following NAMs were considered to be in the scope of RE-Place:

- *In vitro* and *ex vivo* methods (e.g., experiments with the use of 2D and 3D cell lines and tissue cultures, …);
- *In silico* modeling (e.g., molecular modeling and mathematical approaches such as [Quantitative] Structure-Activity Relationships, read across, artificial intelligence, …);
- *In chemico* techniques (e.g., assays without the use of cells or tissues evaluating the reactivity and properties of substances or components, physico-chemical data, …);
- Alternative *in vivo* models (e.g., fruit flies, flatworms, early stages of zebrafish embryo’s, …);
- Imaging techniques (e.g., cellular imaging techniques, or imaging with a clear application to the replacement of laboratory animals);
- High-throughput testing strategies and omics techniques (e.g., genomics, metabolomics, proteomics and transcriptomics) and
- Other innovative techniques (e.g., stem cell technology used in organ-on-a-chip, …).

In this manuscript, the RE-Place project and more in particular, the development of the RE-Place online tool and database are described. Also, an overview of the current status of the database is provided together with some of the encountered challenges and the added value of RE-Place in general.

## 2 Materials and Methods

### 2.1 Development of the RE-Place “Online Tool”

The existing template of DB-ALM^7^ was used as a starting point to determine the type and amount of information to be collected within the scope of the RE-Place project. The DB-ALM is a public database from the EC, more in particular from EURL ECVAM, which was launched about 20 years ago to collect and provide information on alternative methods to animal testing in biomedical sciences and toxicology. The data included in the DB-ALM were collected from literature, validation activities of EURL ECVAM, EU projects and other information sources. The DB-ALM template was quite elaborate and contained questions regarding the protocols used, the tested compounds and the obtained test results. Detailed information on the development and the validation stages were requested as well.

However, as DB-ALM initially targeted methods for future validation, this template was highly focused on technical aspects and was thus too detailed to be used as a basis for RE-Place. Therefore, the requested information was reduced significantly with a focus on collecting only those elements that enable scientists from diverse disciplines to understand the scientific rationale behind the method and to help them to decide whether the NAM could be of interest or not. These “essential elements” include, amongst others, a short description, keywords, laboratory equipment, regulatory status, etc.

Since the entry of information is fully voluntary, the submission process had to be as simple and fast as possible. Using a Word template, similar to the one of DB-ALM, would be very cumbersome and time consuming. Therefore, a more user friendly web-based application was created to collect the information of interest, the so called “RE-Place online tool.” Although the RE-Place project is a regional initiative, the online tool was developed in English as English is generally used by the scientific community. Moreover, this enables users to copy paste information from protocols and/or publications directly into the online tool, further reducing the workload.

Before being launched to the public, the submission process with the RE-Place online tool was tested, analyzed, and evaluated by experts from various institutes during a pilot study. These experts also provided feedback on the type of information requested (i.e., “What is essential information?” and “Which type of requested information is (not) needed in the scope of RE-Place?”). At least two feedback rounds were organized. Based on the feedback received, the submission process was further fine-tuned and the user-friendliness was optimized resulting in the finalized version of the RE-Place online tool. A detailed description of the requested information per step is displayed in Table 1.

**TABLE 1:** Detailed description of the requested information in the different steps of RE-Place online tool.

Step	Topic per step	Further details on the type of requested information	Mandatory or optional
0	Introduction for new users	n/a	*n/a*
1	General information	Title of the method, acronym, organisations linked to the method, groups links to the method, partners linked to the method	*Mandatory*
2	Scope of the method	Area of expertise, type of method	*Mandatory*
3	Description	Keywords associated with the method and scientific area, short description, lab equipment, regulatory status	*Mandatory*
4	Pros, cons and future potential	Advantages, challenges, potential modifications, future and other applications	*Optional*
5	References, associated documents and other information	References, possibility to upload associated documents (such as protocols and publications), external links, other remarks or relevant information	*Optional*
6	Privacy	Contact name disclosure, organisation name disclosure, possibility to notify peers, colleagues, and others about the submitted information	*Mandatory*

After providing some general information with respect to the name and acronym of the method, the user has to select the category/ies to which the method belongs:

- “Basic research”;
- “Translational and applied research”;
- “Regulatory use and routine production”;
- “Education and training”;
- “Other.”

These categories are similar to those in the Member States (MS) national statistical reports on the use of laboratory animals for scientific purposes of the EU^8^. As the area of expertise is often not limited to only one of the defined categories, scientists are able to link their method with multiple areas.

Methods can also be further categorized *via* the following dropdown list:

- *“In vitro* and *ex vivo”:* these two types of methods are combined into one category as the distinction between both is not always clear;
- *“In silico”* or computer modelling techniques;
- *“In vivo”: in vivo* does not refer to actual laboratory animal experiments, but to the use of alternative *in vivo* models such as C. elegans, D. melanogaster, and early stages of a zebrafish embryo;
- *“In chemico* assays”: assays without the use of cells or tissues evaluating the reactivity and properties of substances or components;
- “Other.”

Scientists can also indicate the (regulatory) status of their method. This information allows scientists to better understand in which part of the development or validation phase the method is situated. This categorization is optional and comprises following items:

- “Still in development”;
- “History of use”;
- “Internally validated” (for example, after performing in house validation studies, determining internal criteria for quality control, and defining relevant standards);
- “Published in peer reviewed journal”;
- “Currently submitted for further validation by an external party (e.g., OECD, EURL ECVAM, …)”;
- “Validated by an external party (e.g., OECD, EURL ECVAM, … ).”

Certain information fields of the RE-Place online tool are mandatory (e.g., keywords, short description, … ), so the ‘basic information’ on the described methodologies is available for scientists consulting the RE-Place database. None of the information fields require any type of sensitive information in relation to method development, so the IP rights cannot be violated. Moreover, the majority of the requested information is an “open text field,” meaning that each expert is free to determine the type and amount of information he/she wishes to disclose. For example, if an expert wants to share a full standard operating procedure (e.g., after the patent has expired) this is possible, but not obligatory.

In view of facilitating networking activities and cross-collaborations, personal information (names and e-mail addresses) of the experts who submitted their expertise and the names of the involved (research-) institute(s) are also requested. However, in order to be in line with the General Data Protection Regulation (The European Parliament and the Council of the European Union, 2016), the RE-Place online tool has been designed in such a way that every expert can choose whether his/her personal details are disclosed in the RE-Place database. This preference can be changed at any given time point. In case an expert indicates his/her personal details cannot be disclosed, the RE-Place team will serve as an intermediate contact to transfer questions.

### 2.2 Quality Control and Assurance

In order to guarantee the quality of the submitted information, registration is mandatory, but free of charge, and a short “validity check” on the submitted methods is performed by the RE-Place team via a number of preset requirements. More specifically, 1) the submitted method should be a NAM in the scope of the RE-Place project, 2) the identity of the user and organization(s) need to be legitimate, and 3) the language and lay-out need to be correct.

Information can also be submitted via the features “My Group” and/or “My Organisation,” ensuring additional internal quality controls by the organisation(s) of the expert(s). The feature “My Groups” is based on a role based system and allows supervision by the lead of a particular (research-) group. There can be two roles in a so called “Group” i.e., “Administrators” and “Members.” “Administrators” can review and modify all submitted content linked to their Group, and “Members’ can submit information. In practice, “Administrators” are mainly senior experts, professors, promotors, principle investigators,… while “members” include all scientific and laboratory staff. The feature “My Organisation” allows a better control and follow-up of the accounts and research groups linked to a particular organisation.

### 2.3 Integration of the Collected Information in an Open Access Database

The RE-Place online tool was designed in such a way that all collected information is automatically integrated in the RE-Place open access database. User-friendliness and an attractive lay-out were key attention points in the development process and to this extent, following items were considered:

(i) a modern and intuitive lay-out when browsing the database,
(ii) a clean interface with an emphasis on the search box to facilitate launching queries *via* keywords and pre-set filters (i.e., type of method, area in which the method is situated, involved organisation),
(iii) a structured overview of the obtained search results after launching a query,
(iv) a clear association between the title of a method, the name of an expert, and the corresponding (research-) institute where the expertise is allocated.

### 2.4 Development of an Overarching Structure, the RE-Place Project Website

The RE-Place online tool and open access database have both been embedded in the overall project website: www.RE-Place.be. This website was built with “Drupal 8,” a free and open-source “Content Management System” which allows to quickly build websites, and develop more complex and customized web based applications.

## 3 Results

### 3.1 The RE-Place Platform

**FIGURE 1:**
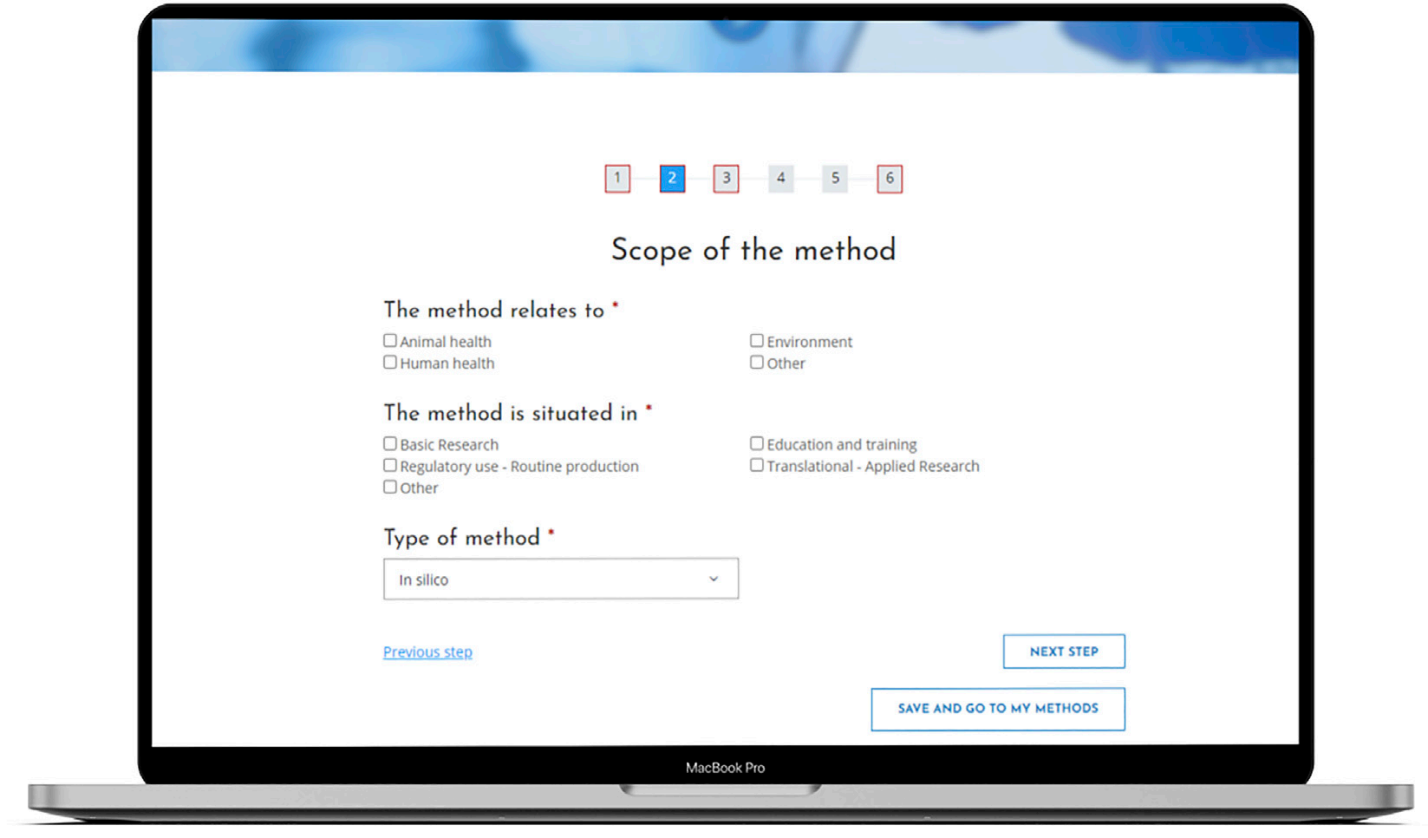
The RE-Place online tool and more in particular, the second step in the submission process.

Figure 1 illustrates the RE-Place online tool. A progress bar is displayed on top of the screen, indicating how far one has advanced in the submission process. The title of each step is shown directly below this bar. One can easily navigate through the online tool by clicking on the corresponding numbers representing the different steps of the submission process or by simply clicking on the button “Next step.”

Steps including mandatory information are indicated by a red square lining. Once all mandatory information has been completed, the red lining will disappear. The blue filling of the numbers indicates where exactly one is situated in the submission process. In Figure 1, the second step of the online tool is represented.

All submitted information is automatically saved, so if one abruptly closes the browser in the middle of the submission process, the information remains available in the online tool. This was a specific request from the experts who evaluated the online tool.

**FIGURE 2:**
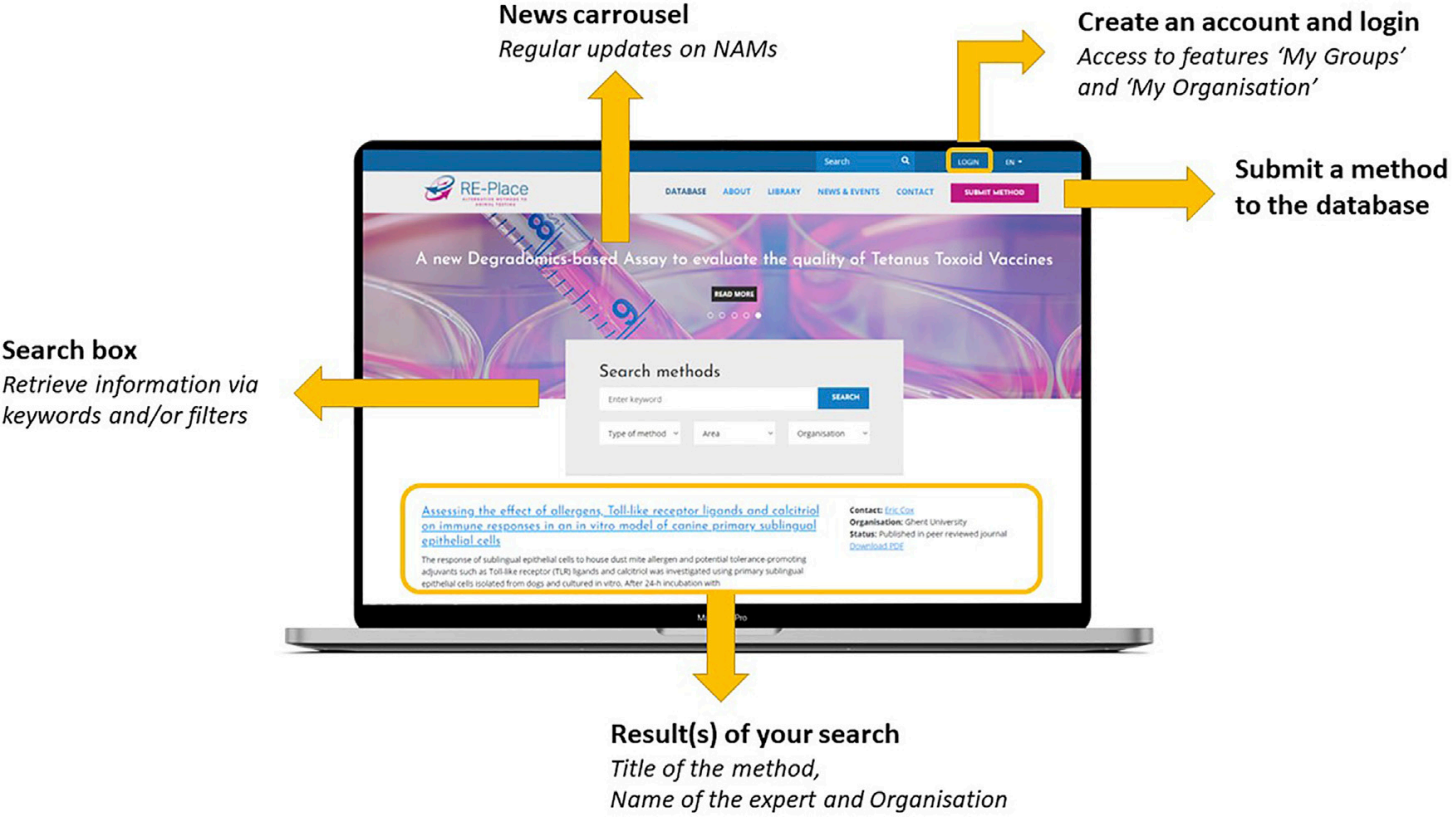
The RE-Place platform with its functionalities.

The RE-Place online tool and database are both fully embedded in the RE-Place website/platform as shown in Figure 2. As the open access database is the most relevant item of the RE-Place project, it is shown as the homepage of the website. One can easily browse through the database by using keywords and/or by applying one of the pre-set filters (i.e., type of method, area in which the method is situated, involved organisation). A Boolean search by inserting “AND” and “OR” functions is also possible, but will be further improved. After launching a query, the obtained results are displayed directly under the search box. The most recent and/or last modified entries are displayed on top of the overview. The date of the last modification is clearly indicated per individual method. Every displayed result provides the title of the method, a teaser of the short description, the name of the expert who submitted it, the name of the organisation where the expert is working and the regulatory status of the method. The names of the experts and organisations are only displayed when consent is given. Access to a detailed overview of each method is obtained by clicking on the method title or by downloading the corresponding PDF.

Within the RE-Place website, additional tabs have been integrated containing more information about:

- The RE-Place project itself: “About”
- General background information about the 3Rs Principle, (inter-)national regulations, useful links: “‘Library”
- Updates on NAMs *via* the news carousel and relevant upcoming events: “News & Events,”
- Information about the RE-Place team and steering committee: “Contact,”
- The most Frequently Asked Questions page “FAQ.”

These tabs, also shown in Figure 2, were translated in Dutch and French to facilitate and increase access of information to the public and other local communities in Belgium.

### 3.2 Status RE-Place Database in May 2022

In May 2022, the RE-Place database contained 193 methodologies; the majority of which was collected between 2019 and 2021. Only a limited number of methods was collected in 2020 and 2021 as due to the COVID-19 pandemic, most congresses were cancelled and onsite presentation of RE-Place in scientific institutes in the Flemish and Brussels region was not possible. Previous experience has taught that these onsite presentations were one of the cornerstones to directly motivate scientists to collaborate. Alternatives were foreseen (video presentations and online meetings), but were far less effective. Although an important number of NAMs has thus already been collected, the current inventory is not yet a full representation of the available expertise on NAMs in Belgium. Within the upcoming years, additional efforts will be undertaken to collect more expertise such as the launch of a promotional video to increase the visibility of the project.

The 193 methods of the RE-Place database were submitted by 132 registered experts from 23 different organisations (including universities, scientific institutes, industry,….) in Belgium. As the “Vrije Universiteit Brussel” (VUB) is one of the coordinators of the RE-Place project, it is not surprising that a large percentage of the available methods (±30%) was submitted by scientists of this university.

An in depth, manual analysis of the keywords used per individual method revealed that a large number of the submitted NAMs is situated in the field of toxicology. Used keywords included, amongst others, (eco)toxicology, acute vs. chronic toxicity, genotoxicity, mutagenicity, (regulatory) toxicology, *in vitro* toxicology, cytotoxicity, hepatotoxicity, toxicogenomics, etc. This can be explained by the fact that a lot of work to replace animal testing was done in this area (Roi and Kolar, 2019), and scientists working in this field are thus more familiar with the topics “3Rs” and “NAMs.” Examples include the SEURAT project, EU-ToxRisk and the ASPIS cluster (Vinken et al., 2021).

**FIGURE 3:**
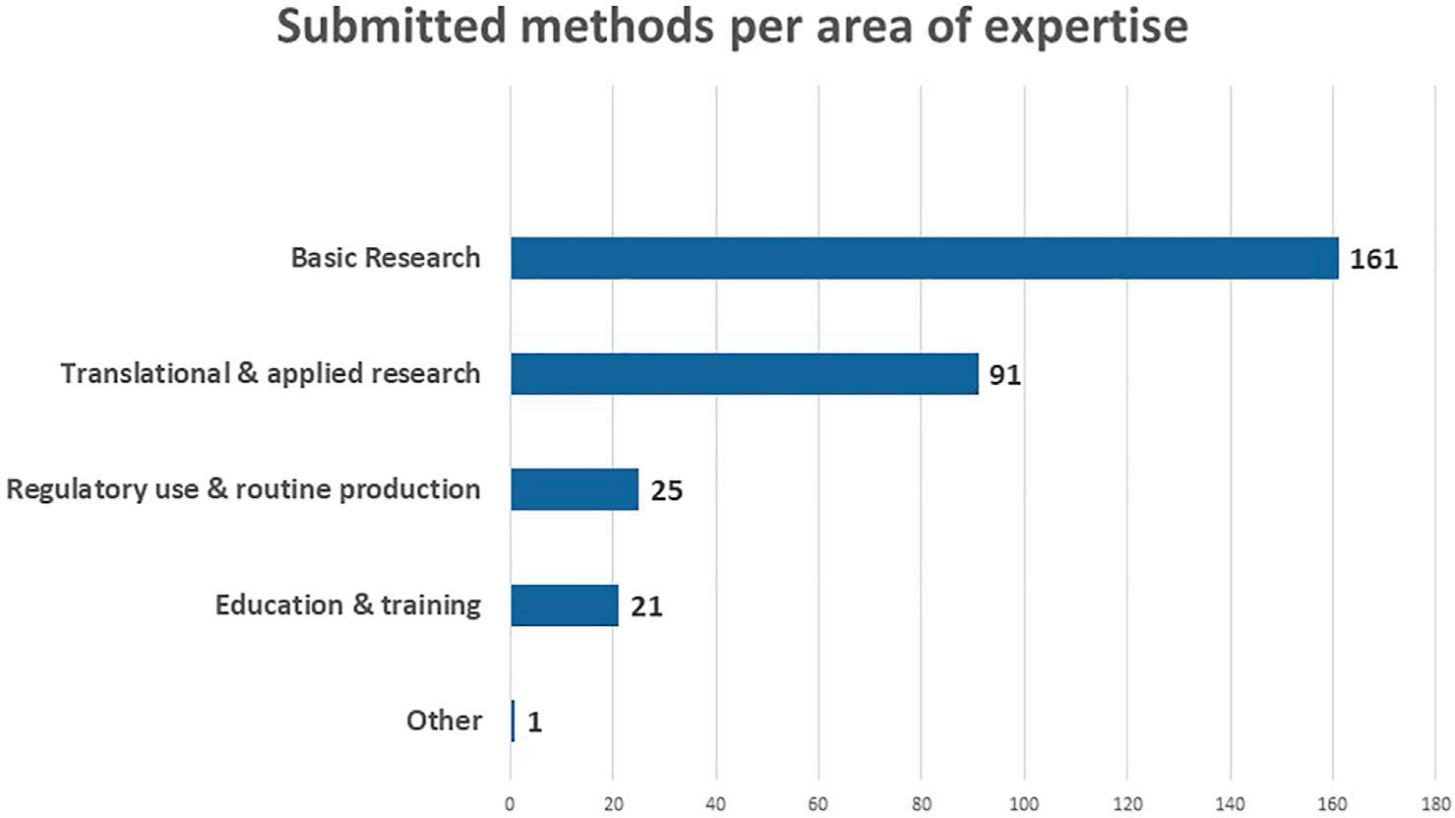
Submitted methods according to the area of expertise in May 2022.

An overview of the submitted methods per area of expertise is provided in Figure 3. 161 of the submitted methods are situated in ‘Basic Research’, followed by 91 in “Translational and Applied Research.” This probably relates to the fact that the RE-Place team mainly focused on contacting experts in biomedical research as there are a lot of opportunities to increase the use of NAMs in this area. In addition, expertise was captured in “Regulatory use and Routine Production” (25) and in “Education and Training” (21). The one method categorized as “Other” is submitted by a scientist working in industry who specified it as “Safety Pharmacology.” This method is also linked to “Translational and Applied Research” and “Regulatory use and routine production.”

**FIGURE 4:**
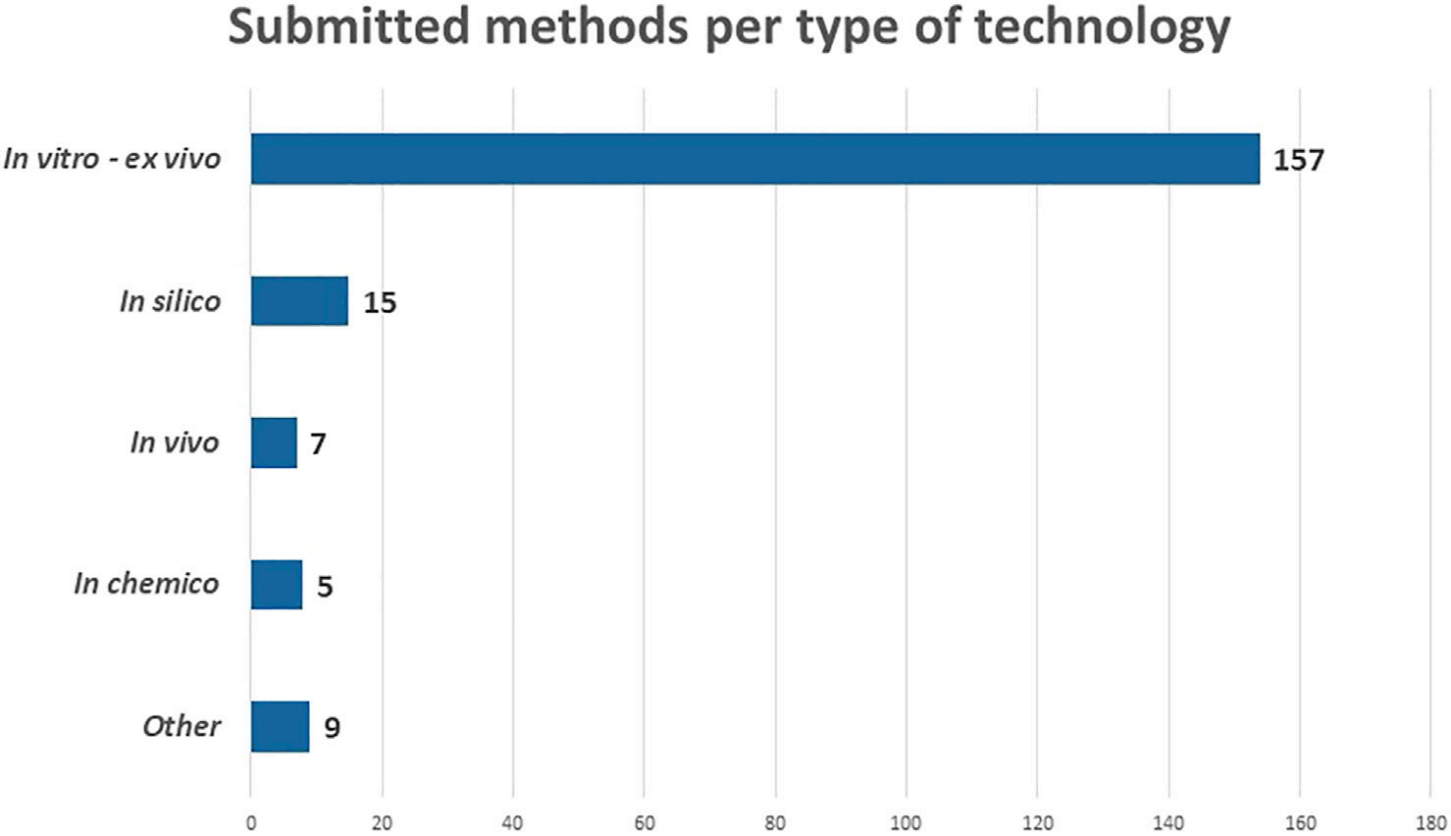
Submitted methods according to the type of technology in May 2022.

An overview of the number submitted NAMs per category is provided in Figure 4. Most methods belong to the category “*in vitro* and *ex vivo”* (157). This is not surprising as a lot of progress in 2D and 3D cell- and tissue cultures was made over the past decade(s), especially since the scientific breakthrough of induced pluripotent stem cells by Shinya Yamanaka in 2006 (Takahashi and Yamanaka, 2006b).

The other types of technologies are far less represented in the RE-Place database. It is, however, expected that the number of “*in silico”* methods (15) will increase as computer modelling techniques and artificial intelligence are rapidly evolving. A possible explanation as to why this category is far less represented at the moment is that the developers are often situated in specific niche domains such as bio-informatics and programming, and might thus not be familiar with the terms “alternatives” and “NAMs.” Seven methods were submitted in the “*in vivo”* category. These include the use of *C. elegans*, *G. mellonella*, *A. castellanii* and fertilized chicken embryos. Finally, five methods were categorized as “*in chemico*,*”* of which three are related to the field of–omics, which are high-throughput biochemical assays, also an emerging technique in the life sciences. Of the other nine remaining NAMs, six were related to (virtual) imaging/monitoring and three to education and training.

**FIGURE 5:**
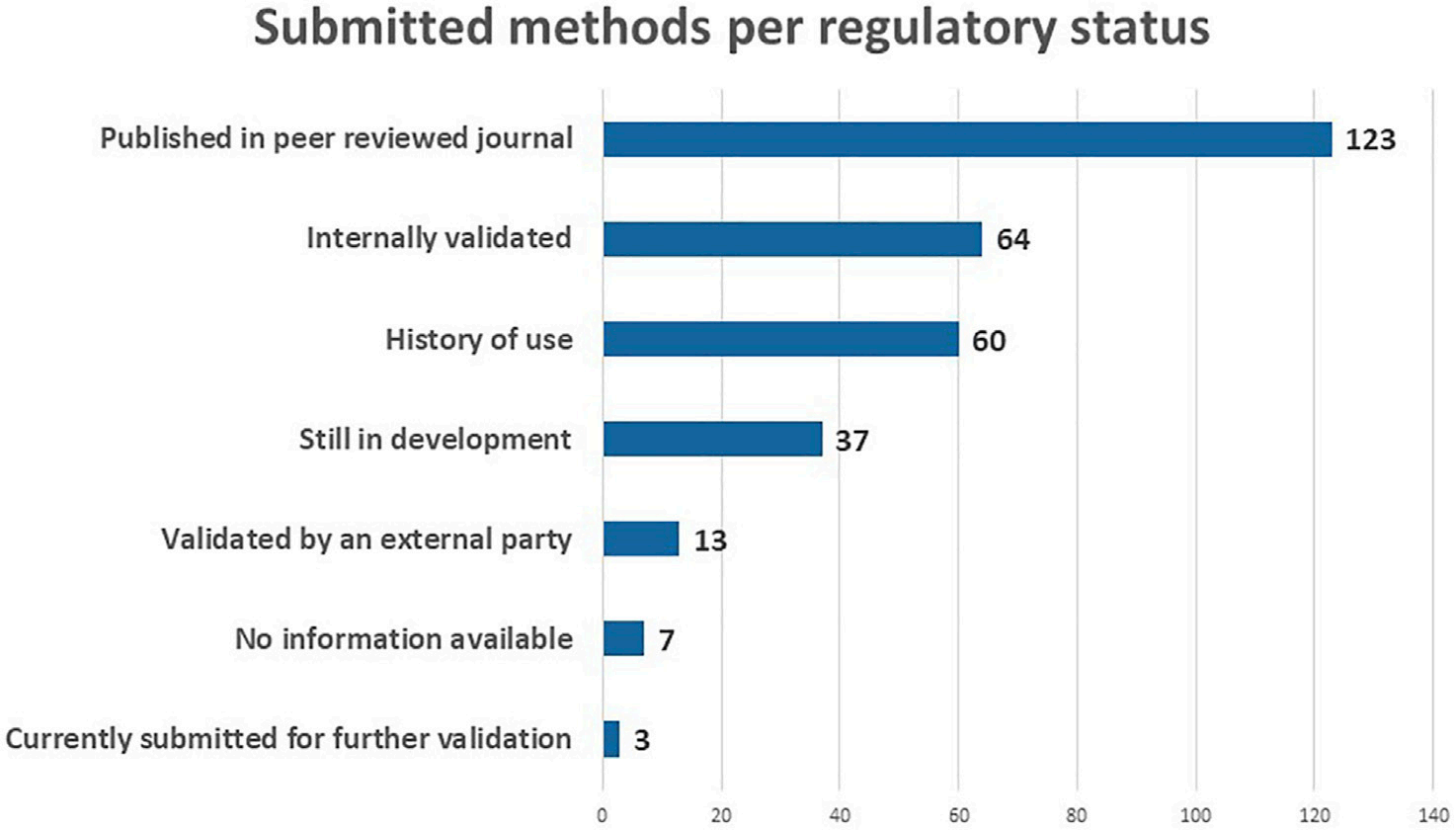
Submitted methods per regulatory status in May 2022.

With respect to the regulatory status, the majority of the submitted methods has been published in a peer reviewed journal (123), meaning that the methods have been evaluated by other experts in the field (Figure 5). A large number of the methods are internally validated (64) and/or have a history of use (60). An advantage of the RE-Place database is that it also collects NAMs which are still under development (37). This allows scientists to learn more about new technologies emerging in the life sciences, and get a better understanding thereof. Lastly, a number of validated NAMs is also included in the database (13), in addition to a small number submitted for further validation (3). Seven methods are not linked to any tye of regulatory status. These categories will help regulators determine which NAMs can already be used to replace animal testing.

Within the RE-Place online tool, both keywords linked to the method/technology itself and to the scientific area in which the NAM is used can be provided. As such, scientists working on similar methodologies, but in different life science areas can connect more easily with each other. However, some issues with the provided keywords were encountered. For example, keywords were sometimes added in both categories or obvious keywords were overlooked. Moreover, the taxonomy in Drupal software does not recognize synonyms. It is thus very difficult to analyze the use of keywords in this system, especially since the software does not indicate how many times a particular keyword was used.

## 4 Discussion

### 4.1 Challenges

#### 4.1.1 Delineating the Scope

Some of the submitted methods were considered outside the scope of the RE-Place project such as for example, micro-dosing and human biomonitoring. Although these methods avoid the direct use of animals, they were not included as they would make the content of the database too diverse. Also methods rather linked to reduction and refinement of animal testing, such as animal or tissue sharing, are at this stage withheld from the database. Methods from other MS without a clear contact person in Belgium are not (yet) included either as at present, the RE-Place database still focuses on the available expertise in Belgium. However, in the future, the scope can be extended to other MS. Finally, NAM-related expertise from industry which has the sole goal to “sell or promote” particular products, is also considered outside the scope of the project unless the methodology can be linked to a scientist who is actually using the method in Belgium.

#### 4.1.2 Identifying Expertise

Identifying Belgian scientists who have expertise in NAMs was also quite challenging. The first and most obvious hurdle is the high turnover rate in personnel, especially at universities where PhD or Master students often leave when their thesis is finalized and knowledge on a particular NAM might be lost. A second one relates to the fact that new technologies might not directly or necessarily replace animal testing, as they can originate from a new out-of-the-box sort of thinking. The scientists involved might therefore not be aware that they are experienced in a methodology which can contribute to the ultimate replacement of animal tests. Consequently, they do not identify themselves nor their work with the terms “alternatives,” “NAMs” and/or the “3Rs.” In order to overcome this issue, scientific literature is regularly reviewed for new keywords and technologies, and scientists are contacted pro-actively to inform them about the RE-Place project. It should, however, be noted that this is a very time consuming task.

#### 4.1.3 Convincing Scientists to Collaborate and Creating Concrete Incentives

One of the main challenges of RE-Place is how to motivate scientists to submit their expertise. When the RE-Place project was launched in 2017, scientists were reluctant to collaborate as the project was rather unknown. In order to increase trust in the project, promotional material was developed and on site information sessions were organized. During these sessions, the possible benefits of contributing to RE-Place were emphasized such as increased visibility of the existing expertise and opportunities to engage in new collaborations. Moreover, a steering committee consisting of 3R experts from various institutes and disciplines in Belgium was established at the start of the project. Having a direct point of contact within an organization generally increased the willingness of the employees within that organization to collaborate. Overall, personal involvement turned out to play a key role in building confidence.

But even a personal contact was not always sufficient to stimulate scientists to submit their methods, as their workload is generally high. Although efforts have been made to minimize the time needed to submit the information needed (i.e., five to 15 minutes), time thus often remains the limiting factor. Stronger incentives are therefore needed such as the possibility to obtain additional funding. However, this was not possible for RE-Place. Nevertheless, a successful incentive was the publication of a Special Issue in MethodsX, combining nine methodologies from the RE-Place database focused in the field of toxicology (Van Mulders et al., 2020). Similar incentives will be further explored in the coming years.

Another hurdle in convincing scientists to collaborate was IP rights. In both the development and validation process of NAMs, protection of sensitive information might be crucial in the scope of a possible future commercialization. Scientists might thus be reluctant to submit their know-how, and that is why RE-Place ensures to avoid any form of infringement of IP rights. It should, however, be noted that data sharing and mutual acceptance of data are key elements in the regulatory uptake and acceptance of methods (Linge and Hartung, 2007) (Ramirez et al., 2015).

#### 4.1.4 Building Bridges

Over the past decades, especially in the Belgian (popularized) media, animal testing has been put as a gold standard on the one side versus the use of NAMs on the other side. The majority of scientists, however, do not agree with this approach, and point out that they are trying to incorporate NAMs wherever scientifically possible. Due to this polarization, researchers are sometimes reluctant to collaborate as they do not want to be associated with or appointed to one particular side: not on the “animal side” nor on the “NAM side.” To avoid further polarization, clear communication on what is (not) (yet) possible with NAMs is essential. In addition to the RE-Place database, accurate and up-to-date information is therefore also included in the RE-Place website. Moreover, personal contact and face-to-face meetings also help to have more nuanced discussions.

#### 4.1.5 Ensuring Long Term Funding

Another major challenge is ensuring the means to fund the RE-Place project on the long-term. At present, funding of RE-Place in ensured until 2024. However, as for any other platform on 3Rs, long term financial investments are needed to keep the database live and up-to-date (Roi and Kolar, 2019) (Holley et al., 2017), more specifically to 1) update the Drupal software, 2) engage personnel, 3) develop and distribute promotional material, 4) organize on site visits to different (research-) institutes in Belgium and 5) take contact with all relevant stakeholders.

### 4.2 Added Value

One could argue about the added value of “another database” as several initiatives to disseminate information on NAMs (e.g., DB-ALM and ZEBET) already exist. Nonetheless, the report of EURL ECVAM indicated more open access databases are needed in which data is collected in a harmonized way (Holley et al., 2017). In order to accelerate the implementation of NAMs, communication is key, and one needs to have ready access to up-to-date, reliable information (Roi and Kolar, 2019) (Ramirez et al., 2015) (Mondou et al., 2021). As discussed above, personal contact is important to convince scientists to disseminate information, and therefore local initiatives such as RE-Place have a clear added value. Moreover, RE-Place includes information on the expert and/or the institute where a technique can be learnt, which makes it unique in that aspect.

As RE-Place centralizes the available expertise on the use of NAMs across disciplines and life science areas in Belgium, it can be used by many different stakeholders:

- Scientists to connect with peers from different research groups and disciplines to exchange experience, enabling a cross-sectorial knowledge transfer. By stimulating communication on NAMs, the confidence in these new technologies can be enforced, accelerating the optimization of the development and/or validation thereof.
- Ethical committees as a practical tool when evaluating research proposals which require the use of experimental animals. By consulting RE-Place, they will have a better understanding in which areas partial or full replacement is (not yet) possible.
- Regulators to learn more about the most recent technologies, how they are used and their potential regulatory relevance.
- Authorities, policy makers and funding bodies to gain a better understanding of what is scientifically feasible with the use of NAMs and to identify knowledge gaps, resulting in a more targeted allocation of funding. For example, one could decide to allocate certain budgets to methods which are ready for validation, or the opposite, to invest in areas where there are (almost) no alternatives available.

The RE-Place database can also be a used as a tool for organisations to create an overview of the available in-house expertise. Universities generally have a high turnover rate in personnel, and large scientific institutes often have research facilities scattered over multiple locations, making it difficult to map relevant in house expertise hampering internal collaborations and progress in the field of NAMs. Moreover, by inserting the available know-how in the RE-Place database, organisations will be able to demonstrate in which areas they are highly qualified in the use of NAMs which will become increasingly important in relation with EU Directive 2010/63 (Commission of the European Communities, 2010).

Another advantage is that methods from different development and validation stadia are included in the RE-Place database. As a result, knowledge sharing is enforced from the very early stages on, which stimulates the setting up of proper validation requirements for these new technologies and their regulatory acceptance in the long term (Mondou et al., 2021) (Ahr et al., 2008). The importance of this early involvement was emphasized during a workshop of “European Partnership for Alternative Approaches to Animal Testing” (EPAA) (Ramirez et al., 2015). In parallel, promoting cross-sectorial collaborations offer valuable opportunities to exchange knowledge that will contribute to the uptake of NAMs (Takahashi and Yamanaka, 2006b) (Ramirez et al., 2015), as highlighted in the report of the EC “Bridging Across Methods in the Biosciences’ (Carusi et al., 2019).

**FIGURE 6:**
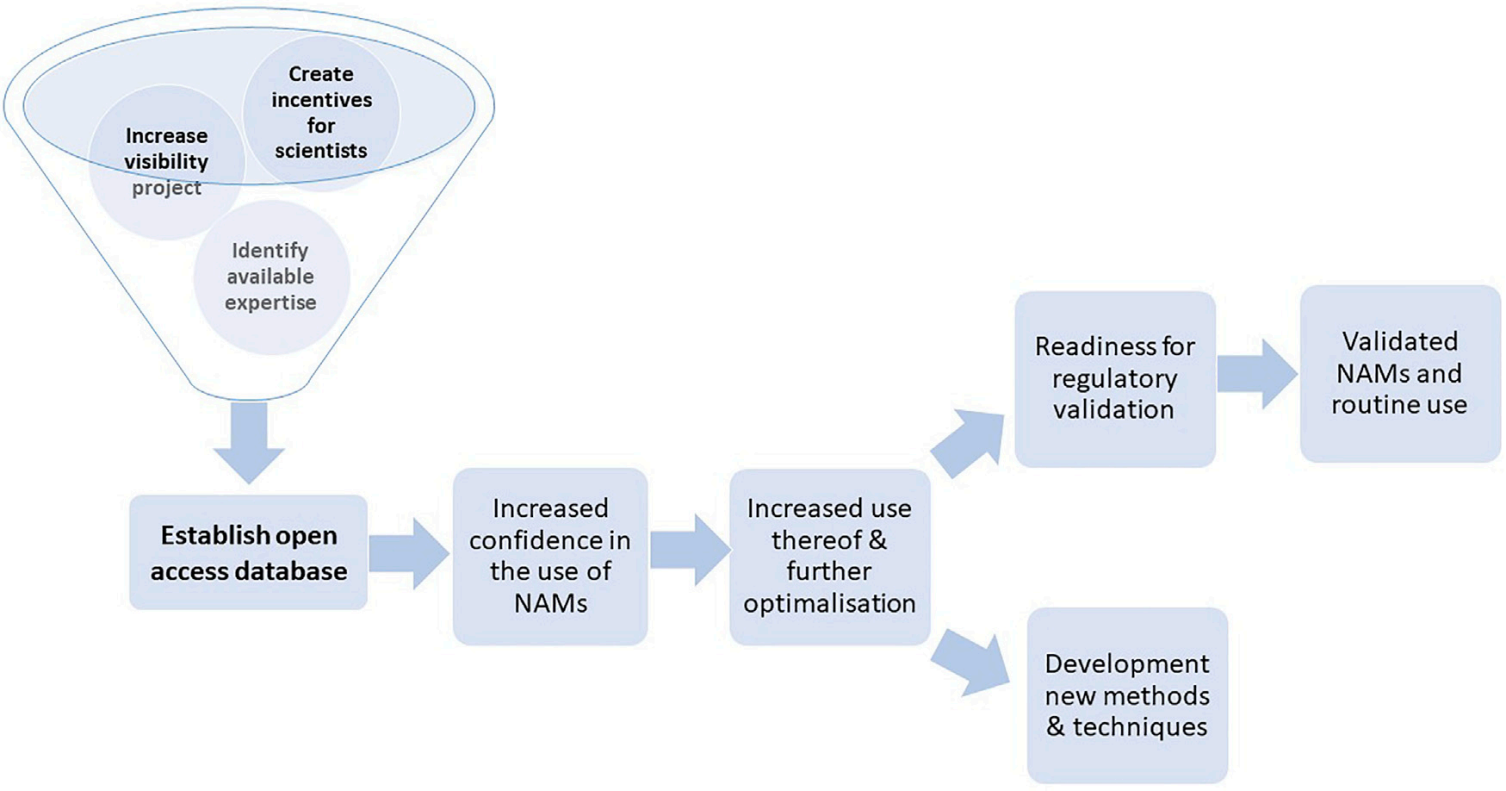
Roadmap to promote the development, use, validation and regulatory uptake of NAMs via the RE-Place project.

A roadmap visualizing the impact of the RE-Place project on the development and validation of NAMs, as described above, is shown in Figure 6.

### 4.3 Next Steps

#### 4.3.1 Possibilities to Broaden the Scope

At present, the RE-Place project is a national initiative which holds particular advantages. For example, it ensures a close personal relationship with the scientific community and other stakeholders in Belgium which will lead to better local networking activities and collaborations. By serving as a focal point of information on NAMs, all relevant stakeholders (scientific community, ethical committees, regulators, government agencies,..) will know directly who to contact for questions in Belgium.

Other MS have already expressed interest in joining the RE-Place database. This could, for example, be done within the context of a European project. However, as the core strength of the RE-Place project is the creation and consolidation of a strong national network, this aspect needs to be considered when extending the RE-Place database. Ideally, each MS should create its own national platform with the support of a local team who establishes and maintains such national contacts. Then, these national platforms could all be linked to one broader platform, offering the best of both worlds. To this extent, it is important that the software used to develop the databases are compatible with each other.

Different possibilities to expand the project will be explored in the coming period and further discussed with the funders of the RE-Place project and the RE-Place steering committee.

#### 4.3.2 Improvements to the RE-Place Platform

Even though the RE-Place platform has been optimized over the past year, there is room for improvement. Several possibilities have already been discussed with the RE-Place steering committee and might be implemented, depending on the remaining budget:

- A more defined system to categorize keywords and taxonomy, which would make it easier to retrieve the right NAM of interest in one search query.
- The possibility to “associate” or “link” NAMs with each other. For example, similarity of keywords could trigger additional suggestions to check related topics.
- The possibility to provide feedback on submitted NAMs would be useful to collect additional information or specific remarks/suggestions on the methods present in the database and could facilitate the actual implementation of NAMs in routine practices. This could be done, for example, via a forum, an option that was already discussed extensively with the RE-Place steering committee and experts in Information Technology. Establishing a forum, however, also holds certain disadvantages. First of all, judging whether or not a specific comment or remark is justified requires in depth knowledge on that specific NAM and even then, this process might be subjective. In addition, allowing a commenting section might result in a high amount of spam messages and counterproductive comments which could impede the (future) use of certain NAMs. An alternative solution would be the use of “a thumbs up” approach to reflect the recommendation of a particular method.
- Grouping of the submitted methods per research area, endpoint or type of technology would be useful, in particular when the database would grow significantly, for example, when it is extended to other MS. In this case, grouping of NAMs per country could also bring an additional value.

The RE-Place project holds thus considerable potential for further expansions and additional improvements. During the further course of the project, it will remain important to carefully consider and evaluate the added value of these changes case by case. The ultimate outcome will also greatly depend on the future (funding) of the project.

## 5 Conclusion

As the development and use of NAMs is a rapidly evolving discipline, it is important to centralize all available expertise and to provide reliable and up-to-date information to the scientific community and other relevant stakeholders. There is a clear need to collaborate across disciplines and life science areas to maximize the true potential of NAMs. The RE-Place database shows that regional/national initiatives can play a key role in promoting the use of NAMs via a bottom-up strategy. Scientists are actively and personally contacted and informed with respect to the RE-Place project and the importance of NAMs. This helps to raise awareness, to identify expertise and knowledge gaps, to stimulate networking activities and to set-up collaborations, all essential elements in promoting the development and use of NAMs. Possible next steps could be setting-up similar initiatives in other MS and/or launching a EU collaboration based on RE-Place.

## Data Availability

The original contributions presented in the study are included in the article/supplementary material, further inquiries can be directed to the corresponding author.

## Footnotes

1
https://data.jrc.ec.europa.eu/dataset/jrc-eurl-ecvam-eurl-ecvam-3rs, accessed 9 December 2020.

2
https://ec.europa.eu/jrc/en/scientific-tool/database-alternative-methods-animal-experimentation, accessed 16 October 2020.

3
https://ntp.niehs.nih.gov/whatwestudy/niceatm/altbib/index.html?utm_source=direct&utm_medium=prod&utm_campaign=ntpgolinks&utm_term=altbib, accessed 6 May 2021.

4
https://apps.bfr.bund.de/animalt-zebet/index.cfm, accessed 11 February 2022.

5
https://norecopa.no/, accessed 16 October 2020.

6
https://www.re-place.be/, accessed 11 February 2022.

7
https://ec.europa.eu/jrc/en/scientific-tool/database-alternative-methods-animal-experimentation, accessed 16 October 2020.

8
https://ec.europa.eu/environment/chemicals/lab_animals/member_states_stats_reports_en.htm, accessed 16 October 2020.
